# Ground state search, hysteretic behaviour, and reversal mechanism of skyrmionic textures in confined helimagnetic nanostructures

**DOI:** 10.1038/srep17137

**Published:** 2015-11-25

**Authors:** Marijan Beg, Rebecca Carey, Weiwei Wang, David Cortés-Ortuño, Mark Vousden, Marc-Antonio Bisotti, Maximilian Albert, Dmitri Chernyshenko, Ondrej Hovorka, Robert L. Stamps, Hans Fangohr

**Affiliations:** 1 Faculty of Engineering and the Environment, University of Southampton, Southampton SO17 1BJ, United Kingdom; 2 SUPA School of Physics and Astronomy, University of Glasgow, Glasgow G12 8QQ, United Kingdom

## Abstract

Magnetic skyrmions have the potential to provide solutions for low-power, high-density data storage and processing. One of the major challenges in developing skyrmion-based devices is the skyrmions’ magnetic stability in confined helimagnetic nanostructures. Through a systematic study of equilibrium states, using a full three-dimensional micromagnetic model including demagnetisation effects, we demonstrate that skyrmionic textures are the lowest energy states in helimagnetic thin film nanostructures at zero external magnetic field and in absence of magnetocrystalline anisotropy. We also report the regions of metastability for non-ground state equilibrium configurations. We show that bistable skyrmionic textures undergo hysteretic behaviour between two energetically equivalent skyrmionic states with different core orientation, even in absence of both magnetocrystalline and demagnetisation-based shape anisotropies, suggesting the existence of Dzyaloshinskii-Moriya-based shape anisotropy. Finally, we show that the skyrmionic texture core reversal dynamics is facilitated by the Bloch point occurrence and propagation.

An ever increasing need for data storage creates great challenges for the development of high-capacity storage devices that are cheap, fast, reliable, and robust. Nowadays, hard disk drive technology uses magnetic grains pointing up or down to encode binary data (0 or 1) in so-called perpendicular recording media. Practical limitations are well understood and dubbed the “magnetic recording trilemma”^1^. It defines a trade-off between three conflicting requirements: signal-to-noise ratio, thermal stability of the stored data, and the ability to imprint information. Because of these fundamental constraints, further progress requires radically different approaches.

Recent research demonstrated that topologically stable magnetic skyrmions have the potential for the development of future data storage and information processing devices. For instance, a skyrmion lattice formed in a monoatomic Fe layer grown on a Ir(111) surface^2^ revealed skyrmions with diameters as small as a few atom spacings. In addition, it has been demonstrated that skyrmions can be easily manipulated using spin-polarised currents of the 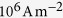 order^3^,^4^ which is a factor 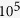 to 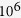 smaller than the current densities required in conventional magneto-electronics. These unique skyrmion properties point to an opportunity for the realisation of ambitious novel high-density, power-efficient storage^5^,^6^ and logic^7^ devices.

Skyrmionic textures emerge as a consequence of chiral interactions, also called the Dzyaloshinskii-Moriya Interactions (DMI), that appear when there is no inversion symmetry in the magnetic system structure. The lack of inversion symmetry can be either due to a non-centrosymmetric crystal lattice structure^8^,^9^ in so-called helimagnetic materials, or at interfaces between different materials that inherently lack inversion symmetry^10^,^11^. According to this, the Dzyaloshinskii-Moriya interaction can be classified either as bulk or interfacial, respectively. Skyrmions, after being predicted^12^,^13^,^14^, were later experimentally observed in magnetic systems with both bulk^15^,^16^,^17^,^18^,^19^ and interfacial^2^,^20^ types of DMI.

So far, a major challenge obstructing the development of skyrmion-based devices has been their thermal and magnetic stability^21^. Only recently, skyrmions were observed at the room temperature in magnetic systems with bulk^22^ and interfacial^23^,^24^,^25^ DMI. However, the magnetic stability of skyrmions in absence of external magnetic field was reported only for magnetic systems with interfacial DMI in one-atom layer thin films^2^,^26^, where the skyrmion state is stabilised in the presence of magnetocrystalline anisotropy.

The focus of this work is on the zero-field stability of skyrmionic textures in confined geometries of bulk DMI materials. Zero-field stability is a crucial requirement for the development of skyrmion-based devices: devices that require external magnetic fields to be stabilised are volatile, harder to engineer and consume more energy. We address the following questions that are relevant for the skyrmion-based data storage and processing nanotechnology. Can skyrmionic textures be the ground state (i.e. have the lowest energy) in helimagnetic materials at zero external magnetic field, and if they can, what is the mechanism responsible for this stability? Do the demagnetisation energy and magnetisation variation along the out-of-film direction^27^ have important contribution to the stability of skyrmionic textures? Is the magnetocrystalline anisotropy an essential stabilisation mechanism? Are there any other equilibrium states that emerge in confined helimagnetic nanostructures? How robust are skyrmionic textures against varying geometry? Do skyrmionic textures undergo hysteretic behaviour in the presence of an external magnetic field (crucial for data imprint), and if they do, what is the skyrmionic texture reversal mechanism?

To resolve these unknowns, we use a full three-dimensional simulation model that makes no assumption about translational invariance of magnetisation in the out-of-film direction and takes full account of the demagnetisation energy. We demonstrate, using this full model, that DMI-induced skyrmionic textures in confined thin film helimagnetic nanostructures are the lowest energy states in the absence of both the stabilising external magnetic field and the magnetocrystalline anisotropy and are able to adapt their size to hosting nanostructures, providing the robustness for their practical use. We demonstrate that both the demagnetisation energy and the magnetisation variation in the out-of-film direction play an important role for the stability of skyrmionic textures. In addition, we report the parameter space regions where other magnetisation configurations are in equilibrium. Moreover, we demonstrate that these zero-field stable skyrmionic textures undergo hysteretic behaviour when their core orientation is changed using an external magnetic field, which is crucial for data imprint. The hysteretic behaviour remains present even in the absence of all relevant magnetic anisotropies (magnetocrystalline and demagnetisation-based shape anisotropies), suggesting the existence of a novel Dzyaloshinskii-Moriya-based shape anisotropy. We conclude the study by showing that the skyrmionic texture core orientation reversal is facilitated by the Bloch point occurrence and propagation, where the Bloch point may propagate in either of the two possible directions. This work is based on the specific cubic helimagnetic material, FeGe with 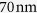 helical period, in order to encourage the experimental verification of our predictions. Other materials could allow either to reduce the helical period^15^,^19^ and therefore the hosting nanostructure size or increase the operating temperature^22^.

Some stability properties of DMI-induced isolated skyrmions in two-dimensional confined systems have been studied analytically^28^,^29^,^30^ and using simulations^26^,^31^. However, in all these studies, either magnetocrystalline anisotropy or an external magnetic field (or both) are crucial for the stabilisation of skyrmionic textures. In addition, an alternative approach to the similar problem, in absence of chiral interactions, where skyrmionic textures can be stabilised at zero external magnetic field and at room temperature using a strong perpendicular anisotropy, has been studied analytically^32^, experimentally^33^,^34^, as well as using simulations^35^. Our new results, and in particular the zero-field skyrmionic ground state in isotropic helimagnetic materials, can only be obtained by allowing the chiral modulation of magnetisation direction along the film normal, which has recently been shown to radically change the skyrmion energetics^27^.

## Results

### Equilibrium states

**Figure 1:**
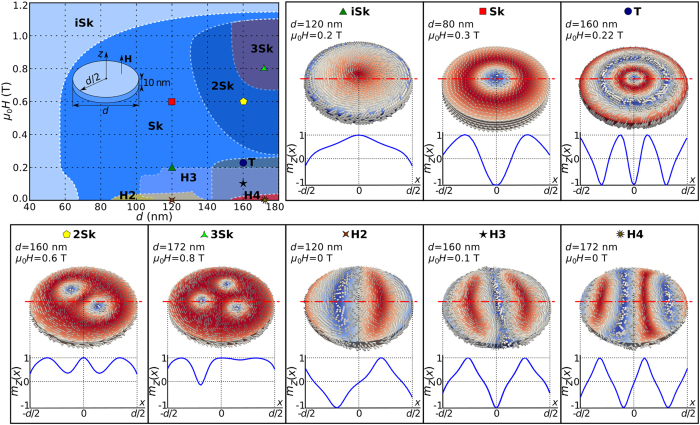
The metastability phase diagram and magnetisation configurations of all identified equilibrium states. The phase diagram with regions where different states are in equilibrium together with magnetisation configurations and out-of-plane magnetisation component 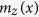 along the horizontal symmetry line corresponding to different regions in the phase diagram.

In order to identify the lowest energy magnetisation state in confined helimagnetic nanostructures, firstly, all equilibrium magnetisation states (local energy minima) must be identified, and secondly, their energies compared. In this section, we focus on the first step - identifying the equilibrium magnetisation states. We compute them by solving a full three-dimensional model using a finite element based micromagnetic simulator. In particular, we simulate a thin film helimagnetic FeGe disk nanostructure with thickness 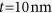 and diameter 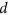, as shown in Fig. 1 inset. The finite element mesh discretisation is such that the maximum spacing between two neighbouring mesh nodes is below 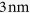. The material parameters are 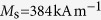, 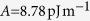, and 
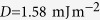
. We apply a uniform external magnetic field perpendicular to the thin film sample, i.e. in the positive 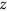-direction. The Methods section contains the details about the model, FeGe material parameters estimation, as well as the simulator software.

In this section, we determine what magnetisation configurations emerge as the equilibrium states at different *d*–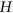 parameter space points. In order to do that, we systematically explore the parameter space by varying the disk sample diameter 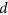 from 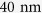 to 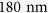 and the external magnetic field 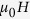 from 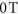 to 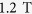 in steps of 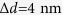 and 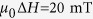, respectively. At every point in the parameter space, we minimise the energy for a set of different initial magnetisation configurations: (i) five different skyrmionic configurations, (ii) three helical-like configurations with different helical period, (iii) the uniform out-of-plane configuration, and (iv) three random magnetisation configurations. We use the random magnetisation configurations in order to capture other equilibrium states not obtained by relaxing the well-defined initial magnetisation configurations. The details on how we define and generate initial magnetisation configurations are provided in the Supplementary Section S1.

The equilibrium states to which different initial magnetisation configurations relax in the energy minimisation process (at every 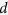
–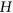 parameter space point) we present in the Supplementary Section S2 as a set of “relaxation diagrams”. We summarise these relaxation diagrams and determine the phase space regions where different magnetisation states are in equilibrium, and show them in Fig. 1. Among the eight computed equilibrium states, three are radially symmetric and we label them as iSk, Sk, and T, whereas the other states, marked as H2, H3, H4, 2Sk, and 3Sk, are not. Subsequently, we discuss the meaning of the chosen labels.

Now, we focus on the analysis of radially symmetric skyrmionic equilibrium states, supported by computing the skyrmion number 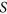 and scalar value 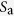 as defined in the Methods section. In the first configuration, marked in Fig. 1 as iSk, the out-of-plane magnetisation component 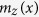 profile along the horizontal symmetry line does not cover the entire 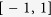 range, as would be the case for a skyrmion configuration (where the magnetisation vector field 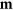 needs to cover the whole sphere). Accordingly, the scalar value 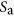 (Eq. (6) in the Methods section, and plotted in Supplementary Fig. 2(b) for a range of configurations), is smaller than 1. For these reasons we refer to this skyrmionic equilibrium state as the incomplete Skyrmion (iSk) state. A similar magnetisation configuration has been predicted and observed in other works for the case of two-dimensional systems in the presence of magnetocrystalline anisotropy where it is called either the quasi-ferromagnetic^26^,^28^ or edged vortex state^29^,^31^. Because the iSk equilibrium state clearly differs from the ferromagnetic configuration and using the word vortex implies the topological charge of 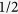, we prefer calling this state the incomplete skyrmion state. The incomplete Skyrmion (iSk) state emerges as an equilibrium state in the entire simulated 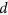
–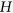 parameter space range. In the second equilibrium state, marked as Sk in Fig. 1, 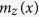 covers the entire 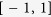 range, the magnetisation covers the sphere at least once and, consequently, the skyrmion configuration is present in the simulated sample. Although the skyrmion number value (Eq. (5) in the Methods section) for this solution is 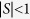 due to the additional magnetisation tilting at the disk boundary^28^, which makes it indistinguishable from the previously described iSk equilibrium state, the scalar value is 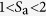. This state is referred to as the isolated Skyrmion or just Skyrmion (Sk), in two-dimensional systems^26^,^28^, and we use the same name subsequently in this work. We find that the Sk state is not in equilibrium for sample diameters smaller than 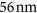 and external magnetic field values larger than approximately 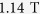. Finally, the equilibrium magnetisation state marked as T in Fig. 1 covers the sphere at least twice. In other works, this state together with all other predicted higher-order solutions (not observed in this work) are called the “target states”^30^, and we use the same Target (T) state name. The analytic model, used for generating initial states, also predicts the existence of higher-order target states (Supplementary Fig. 2(c)). The T magnetisation configuration emerges as an equilibrium state for samples with diameter 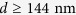 and field values 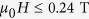.

The equilibrium states lacking radial symmetry can be classified into two groups: helical-like (marked as H2, H3, and H4) and multiple skyrmion (marked as 2Sk and 3Sk) states. The difference between the three helical-like states is in their helical period. More precisely, in the studied range of disk sample diameter values, either 2, 3, or 4 helical half-periods, including the additional magnetisation tilting at the disk sample edge due to the specific boundary conditions^28^, fit in the sample diameter. Consequently, we refer to these states, that occur as an equilibrium state for samples larger than 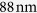 and field values lower than 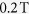, as H2, H3, and H4. The other two radially non-symmetric equilibrium states are the multiple skyrmion configurations with 2 or 3 skyrmions present in the sample and we call these equilibrium states 2Sk and 3Sk, respectively. These configurations emerge as equilibrium states for samples with 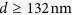 and external magnetic field values between 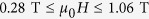.

### Ground state

**Figure 2:**
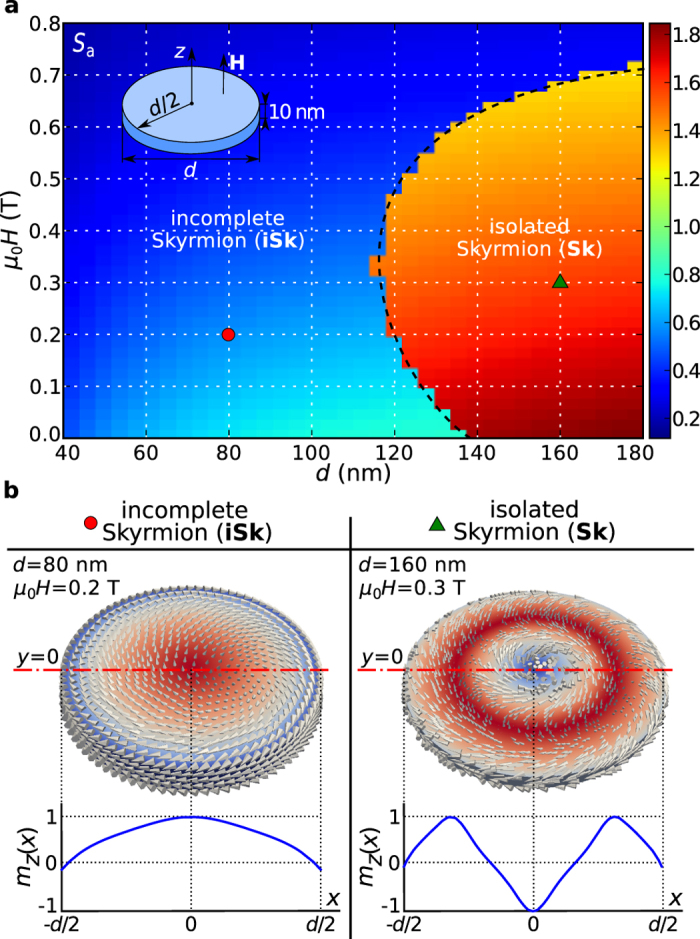
Thin film disk ground state phase diagram and corresponding magnetisation states. (**a**) The scalar value 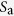 for the thin film disk sample with thickness 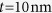 as a function of disk diameter 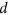 and external out-of-plane magnetic field 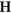 (as shown in an inset). (**b**) Two identified ground states: incomplete Skyrmion (iSk) and isolated Skyrmion (Sk) magnetisation configurations at single phase diagram points together with their out-of-plane magnetisation component 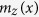 profiles along the horizontal symmetry line.

After we identified all observed equilibrium states in confined helimagnetic nanostructures, in this section we focus on finding the equilibrium state with the lowest energy at all 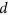
–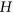 parameter space points. For every parameter space point (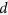, 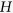), after we compute and compare the energies of all found equilibrium states, we determine the lowest energy state, and refer to it, in this context, as the ground state. For the identified ground state, we compute the scalar value 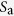 and use it for plotting a 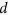
–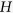 phase diagram shown in Fig. 2(a). Discontinuous changes in the scalar value 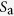 define the boundaries between regions where different magnetisation configurations are the ground state. In the studied phase space, two different ground states emerge in the confined helimagnetic FeGe thin film disk samples: one with 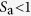 and the other with 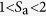. The previous discussion of the 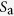 value suggests that these two regions correspond to the incomplete Skyrmion (iSk) and the isolated Skyrmion (Sk) states. We confirm this by visually inspecting two identified ground states, taken from the two phase space points (marked with circle and triangle symbols) in different regions, and show them in Fig. 2(b) together with their out-of-plane magnetisation component 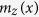 along the horizontal symmetry line.

A key result of this study is that both incomplete Skyrmion (iSk) and isolated Skyrmion (Sk) are the ground states at zero external magnetic field for different disk sample diameters. More precisely, iSk is the ground state for samples with diameter 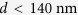 and Sk is the ground state for 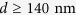. The Sk changes to the iSk ground state for large values of external magnetic field.

**Figure 3:**
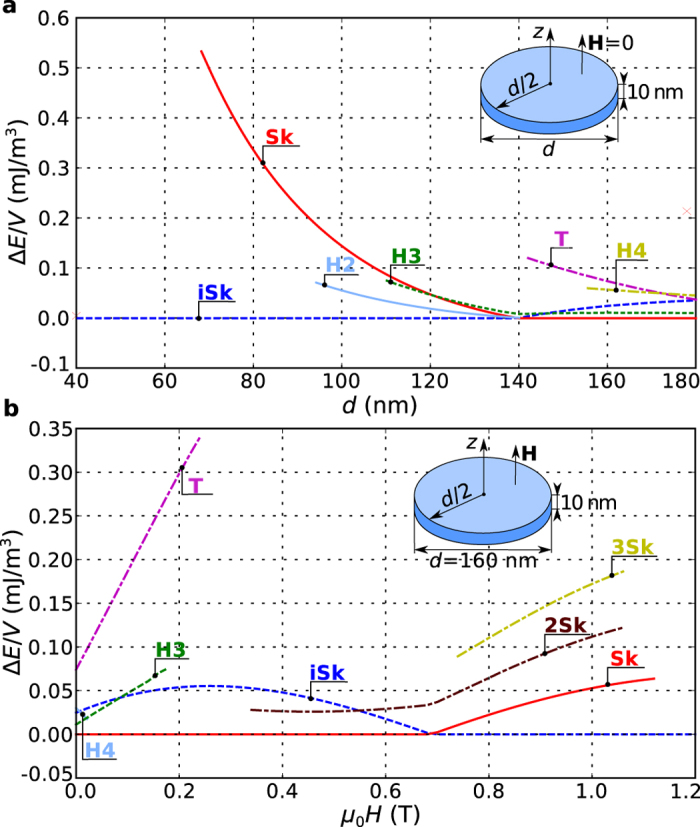
The energy density difference between identified equilibrium states and the corresponding ground state. Energy density differences 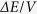 at (**a**) zero field for different sample diameters 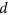 and for (**b**) sample diameter 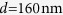 and different external magnetic field values. Configurations are in equilibrium where the line is shown and collapse for other diameter or external magnetic field values.

The phase diagram in Fig. 2 shows the phase space regions where iSk and Sk are the ground states, which means that all other previously identified equilibrium states are metastable. Now, we focus on computing the energies of metastable states relative to the identified ground state. Firstly, we compute the energy density 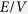 for all equilibrium states, where 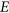 is the total energy of the system and 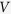 is the disk sample volume, and then subtract the ground state energy density corresponding to that phase space point. We show the computed energy density differences 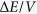 when the disk sample diameter is changed in steps of 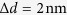 at zero external magnetic field in Fig. 3(a). Similarly, the case when the disk sample diameter is 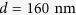 and the external magnetic field is changed in steps of 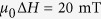 is shown in Fig. 3(b). The magnetisation configurations are the equilibrium states in the 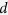 or 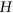 values range where the line is shown and collapse otherwise.

**Figure 4:**
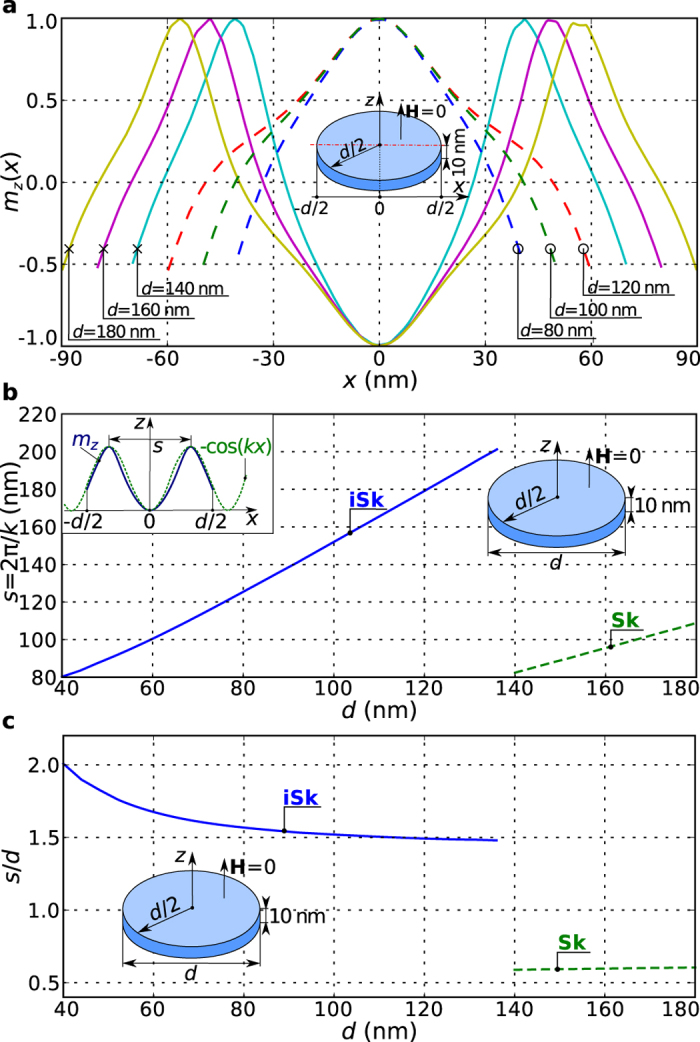
The 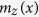 profiles and skyrmionic texture sizes 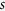 for different sizes of hosting nanostructures at zero external magnetic field. (**a**) Profiles of the out-of-plane magnetisation component 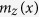 along the horizontal symmetry line for different thin film disk sample diameters with thickness 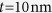 at zero external magnetic field 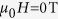. The curves for 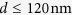 represent incomplete skyrmion (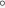) states, and for 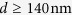 represent isolated skyrmion (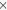) states. (**b**) The skyrmionic texture size 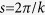 (that can be interpreted as the length along which the full magnetisation rotation occurs) as a function of the hosting nanostructure size, obtained by fitting 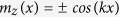 to the simulated profile. (**c**) The ratio of skyrmionic texture size to disk sample diameter (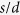) as a function of hosting nanostructure size 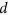.

For the practical use of ground state skyrmionic textures in helimagnetic nanostructures, their robustness is of great significance due to the unavoidable variations in the patterning process. Because of that, in Fig. 4(a) we plot the out-of-plane magnetisation component 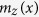 along the horizontal symmetry line for the iSk and the Sk ground state at zero external magnetic field for six different diameters 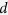 of the hosting disk nanostructure: three iSk profiles for 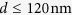, and three Sk profiles for 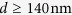. The profiles show that the two skyrmionic ground states have the opposite core orientations. In the case of the Sk states, the magnetisation at the core is antiparallel and at the outskirt parallel to the external magnetic field. This reduces the Zeeman energy 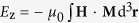 because the majority of the magnetisation in the isolated skyrmion outskirts points in the same direction as the external magnetic field 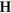. Once the disk diameter is sufficiently small that less than a complete spin rotation fits into the sample, this orientation is not energetically favourable anymore and the iSk state emerges. In this iSk state, the core magnetisation points in the same direction as the external magnetic field in order to minimise the Zeeman energy. We compute and plot the skyrmionic texture size 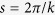 as a function of the disk sample diameter 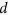 in Fig. 4(b). We obtain the size 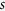, that can be interpreted as the length along which the full magnetisation rotation occurs, by fitting 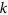 in the 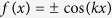 function to the simulated iSk and Sk 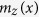 profiles. In Fig. 4(c), we show how the ratio of skyrmionic texture size to disk sample diameter (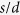) depends on the hosting nanostructure size. Although this ratio is constant (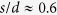) for the Sk state, in the iSk case, it is larger for smaller samples and decreases to 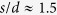 in larger nanostructures. In agreement with related findings for two-dimensional disk samples^29^ we find that both iSk and Sk are able to change their size 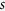 in order to accommodate the size of hosting nanostructure, which provides robustness for the technological use.

**Figure 5:**
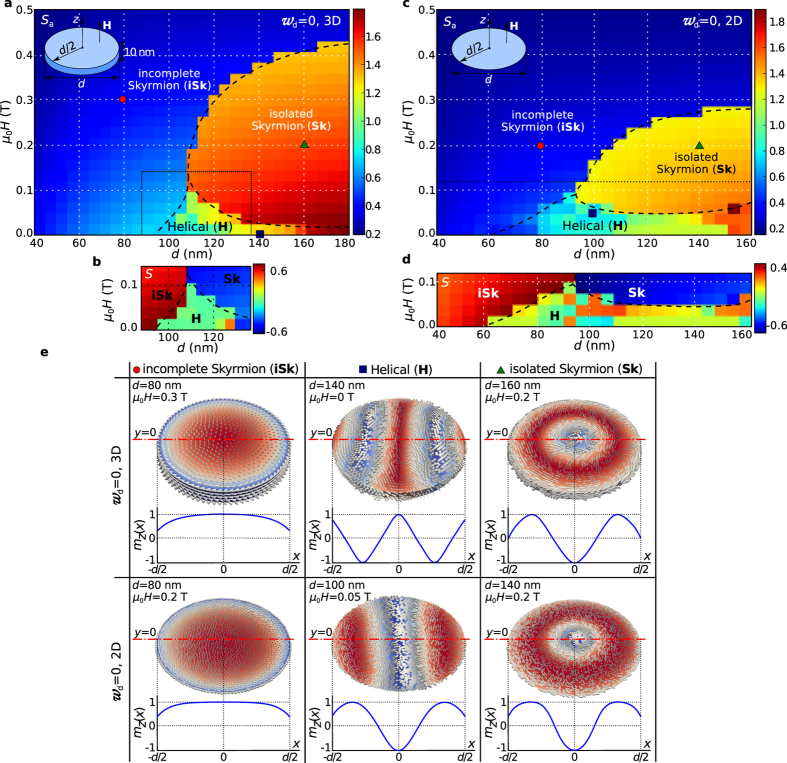
The ground state phase diagram in absence of demagnetisation energy contribution. The scalar value 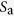 as a function of disk sample diameter 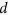 and external magnetic field 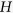 computed for the ground state at every phase space point in absence of demagnetisation energy contribution for (**a**) a 3d mesh and (**c**) for a 2d mesh. In order to better resolve the boundaries of the Helical (H) state region, the skyrmion number 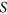 is shown in (**b**,**d**). (**e**) The magnetisation configurations of three identified ground states as well as the out-of-plane magnetisation component 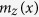 along the horizontal symmetry line.

The emergence of skyrmionic texture ground state in helimagnetic nanostructures at zero external magnetic field and in absence of magnetocrystalline anisotropy is unexpected^21^. Now, we discuss the possible mechanisms, apart from the geometrical confinement, responsible for this stability, in particular (i) the demagnetisation energy contribution, and (ii) the magnetisation variation along the out-of-film direction which can radically change the skyrmion energetics in infinitely large helimagnetic thin films^27^. We repeat the simulations using the same method and model as above but ignoring the demagnetisation energy contribution (i.e. setting the demagnetisation energy density 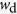 in Eq. (1) artificially to zero). We then carry out the calculations (i) on a three-dimensional (3d) mesh (i.e. with spatial resolution in 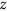-direction) and (ii) on a two-dimensional (2d) mesh (i.e. with no spatial resolution in 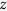-direction, and thus not allowing a variation of the magnetisation along the 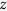-direction). The disk sample diameter 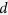 is changed between 40 nm and 180 nm in steps of 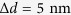 and the external magnetic field 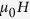 is changed systematically between 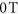 and 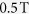 in steps of 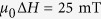. The two resulting phase diagrams are shown in Fig. 5, where subplots (a) and (c) show 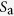 as a function of 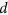 and 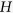. Because the scalar value 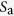 does not provide enough contrast to determine the boundaries of the new Helical (H) ground state region, the skyrmion number 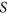 is plotted for the relevant phase diagram areas and shown in Fig. 5(b) and Fig. 5(d).

We demonstrate the importance of including demagnetisation effects into the model by comparing Fig. 5(a) (without demagnetisation energy) and Fig. 2(a) (with demagnetisation energy). In the absence of the demagnetisation energy, the isolated Skyrmion (Sk) configuration is not found as the ground state at zero applied field; instead, Helical (H) configurations have lower energies. At the same time, the external magnetic field at which the skyrmion configuration ground state disappears is reduced from about 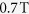 to about 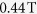.

By comparing Fig. 5(a) computed on a 3d mesh and Fig. 5(c) computed on a 2d mesh, we can see the importance of spatial resolution in the out-of-plane direction of the thin film, and how it contributes to the stabilisation of isolated Skyrmion (Sk) state. In the 2d model, the field range over which skyrmions can be observed as the ground state is further reduced to approximately [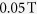, 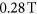]. In the 3d mesh model the Sk configuration can reduce its energy by twisting the magnetisation at the top of the disk relative to the bottom of the disk so that along the 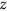-direction the magnetisation starts to exhibit (a part of) the helix that arises from the competition between symmetric exchange and DMI energy terms, similar to Ref. 27. A similar twist provides no energetic advantage to the helix configuration, thus the Sk state region in Fig. 5(a) is significantly larger than the Sk state region in Fig. 5(c) where the 2d mesh does not allow any variation of the magnetisation along the 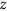-direction and thus the partial helix cannot form.

While the isolated Skyrmion (Sk) configuration at zero field is a metastable state in the absence of demagnetisation energy, or in 2d models, it is not the ground state anymore as there are Helical (H) equilibrium configurations that have lower total energy. The demagnetisation energy appears to suppress these helical configurations which have a lower energy than the skyrmion. The variation of the magnetisation along the 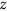-direction stabilises the skyrmion configuration substantially. These findings demonstrate the subtle nature of competition between symmetric exchange, DM and demagnetisation interactions, and show that ignoring the demagnetisation energy or approximating the thin film helimagnetic samples using two-dimensional models is not generally justified.

### Hysteretic behaviour

The phase diagram in Fig. 2(a) shows the regions in which incomplete Skyrmion (iSk) and isolated Skyrmion (Sk) configurations are the ground states. Intuitively, one can assume that for every sample diameter 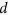 at zero external magnetic field, there are two possible skyrmionic magnetisation configurations of equivalent energy: core pointing up or core pointing down, suggesting that these textures can be used for an information bit (0 or 1) encoding. We now investigate this hypothesis and study whether an external magnetic field can be used to switch the skyrmionic state orientation (crucial for data imprint) by simulating the hysteretic behaviour of ground state skyrmionic textures.

**Figure 6:**
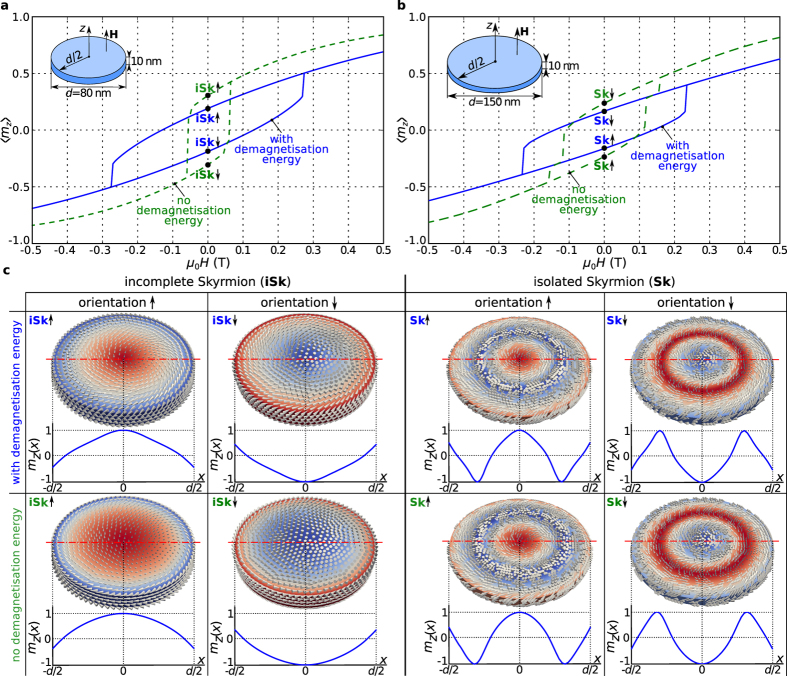
Hysteresis loops and obtained zero-field skyrmionic states with different orientations. The average out-of-plane magnetisation component 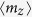 hysteretic dependence on the external out-of-plane magnetic field 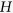 for 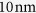 thin film disk samples for (**a**) incomplete Skyrmion (iSk) magnetisation configuration in 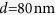 diameter sample and (**b**) isolated Skyrmion (Sk) magnetisation configuration in 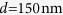 diameter sample. (**c**) The magnetisation states and 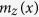 profiles along the horizontal symmetry lines for positive and negative iSk and Sk core orientations from 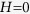 in the hysteresis loop, both in presence and in absence of demagnetisation energy (demagnetisation-based shape anisotropy).

We obtain the hysteresis loops in the usual way by evolving the system to an equilibrium state after changing the external magnetic field, and then using the resulting state as the starting point for a new evolution. In this way, a magnetisation loop takes into account the history of the magnetisation configuration. The external magnetic field 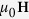 is applied in the positive 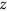-direction and changed between 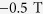 and 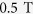 in steps of 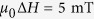. The hysteresis loops are represented as the dependence of the average out-of-plane magnetisation component 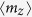 on the external magnetic field 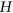. The hysteresis loop for a 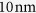 thin film disk sample with 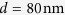 diameter in which the incomplete Skyrmion (iSk) is the ground state is shown in Fig. 6(a) as a solid line. Similarly, a solid line in Fig. 6(b) shows the corresponding hysteresis loop for a larger disk sample with 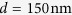 diameter in which the isolated Skyrmion (Sk) is the ground state. The hysteresis between two energetically equivalent skyrmionic magnetisation states with the opposite core orientation at zero external magnetic field, shown in Fig. 6(c), is evident. Moreover, the system does not relax to any other equilibrium state at any point in the hysteresis loop, which demonstrates the bistability of skyrmionic textures in studied system. The area of the open loop in the hysteresis curve is a measure of the work needed to reverse the core orientation by overcoming the energy barrier separating the two skyrmionic states with opposite core orientation.

As throughout this work, it is assumed that the simulated helimagnetic material is isotropic, and thus, the magnetocrystalline anisotropy energy contribution is neglected. Due to that, one might expect that the obtained hysteresis loops are the consequence of demagnetisation-based shape anisotropy. To address this, we simulate hysteresis using the same method, but this time in absence of the demagnetisation energy contribution. More precisely, the minimalistic energy model contains only the symmetric exchange and Dzyaloshinskii-Moriya interactions together with Zeeman coupling to an external magnetic field. We show the obtained hysteresis loops in Fig. 6(a,b) as dashed lines. The hysteretic behaviour remains, although all energy terms that usually give rise to the hysteretic behaviour (magnetocrystalline anisotropy and demagnetisation energies) were neglected. This suggests the existence of a new magnetic anisotropy that we refer to as the Dzyaloshinskii-Moriya-based shape anisotropy.

### Reversal mechanism

The hysteresis loops in Fig. 2 show that skyrmionic textures in confined thin film helimagnetic nanostructures undergo hysteretic behaviour and that an external magnetic field can be used to change their orientation from core pointing up to core pointing down and vice versa. In this section, we discuss the mechanism by which the skyrmionic texture core orientation reversal occurs. We simulate a 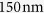 diameter thin film FeGe disk sample with 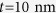 thickness. The maximum spacing between two neighbouring finite element mesh nodes is reduced to 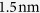 in order to better resolve the magnetisation field. According to the hysteresis loop in Fig. 6(b), the switching field 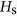 of the isolated skyrmion state in this geometry from core orientation down to core orientation up is 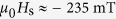. Therefore, we first relax the system at 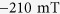 external magnetic field and then decrease it abruptly to 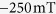. We simulate the magnetisation dynamics for 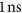, governed by a dissipative LLG equation^36^ with Gilbert damping 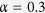
26, and record it every 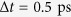.

**Figure 7:**
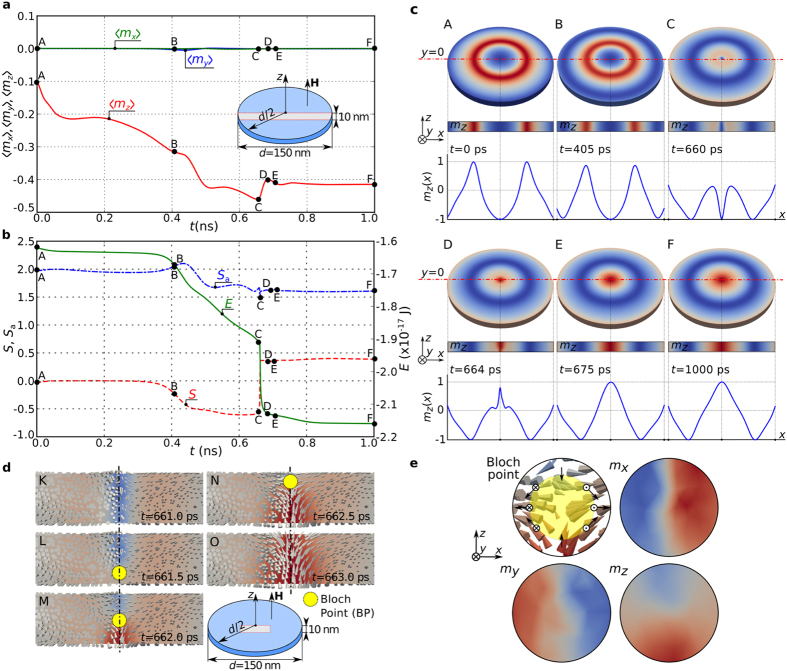
The isolated skyrmion orientation reversal in confined three-dimensional helimagnetic nanostructure. (**a**) The spatially averaged magnetisation components 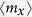, 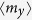, and 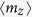 and (**b**) skyrmion number 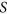, scalar value 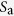, and total energy 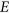 time evolutions in the reversal process over 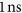. The simulated sample is a 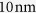 thin film disk with 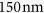 diameter. (**c**) The magnetisation states at different instances of time (points A to F) together with 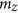 colourmap in the 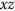 cross section and 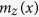 profiles along the horizontal symmetry line. (**d**) The 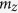 colourmap and magnetisation field in the central part of 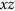 cross section as shown in an inset together with the position of Bloch point (BP). (**e**) The BP structure along with colourmaps of magnetisation components which shows that the magnetisation covers the closed surface (sphere surrounding the BP) exactly once.

We now look at how certain magnetisation configuration parameters evolve during the reversal process. We show the time-dependent average magnetisation components 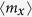, 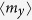, and 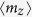 in Fig. 7(a), and on the same time axis, the skyrmion number 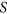, scalar value 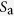 and total energy 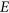 in Fig. 7(b). The initial magnetisation configuration at 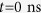 is denoted as A and the final relaxed magnetisation at 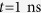 as F. We show in Fig. 7(c) the out-of-plane magnetisation field component 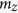 in the whole sample, in the 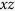 cross section, as well as along the horizontal symmetry line. At approximately 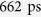 the skyrmionic core reversal occurs and Fig. 7(b) shows an abrupt change both in skyrmion number 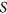 and total energy 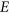. We summarise the reversal process with the help of six snapshots shown in Fig. 7(c). Firstly, in (A-B), the isolated skyrmion core shrinks. At some point the maximum 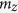 value lowers from 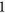 to approximately 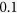 (C). After that, the core reverses its direction (D) and an isolated skyrmion of different orientation is formed (E). From that time onwards, the core expands in order to accommodate the size of hosting nanostructure, until the final state (F) is reached. The whole reversal process is also provided in Supplementary Video 1.

In order to better understand the actual reversal of the skyrmionic texture core between 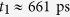 and 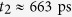, we show additional snapshots of the magnetisation vector field and 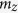 colourmap in the 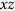 cross section in Fig. 7(d). The location marked by a circle in subplots L, M, and N identifies a Bloch Point (BP): a noncontinuous singularity in the magnetisation pattern where the magnetisation magnitude vanishes to zero^37^,^38^. Because micromagnetic models assume constant magnetisation magnitude, the precise magnetisation configuration at the BP cannot be obtained using micromagnetic simulations^39^. However, it is known how to identify the signature of the BP in such situations: the magnetisation direction covers any sufficiently small closed surface surrounding the BP exactly once^40^,^41^. We illustrate this property in Fig. 7(e) using a vector plot together with 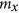, 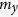, and 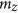 colour plots that show the structure of a Bloch point. We conclude that the isolated skyrmion core reversal occurs via Bloch Point (BP) occurrence and propagation. Firstly, at 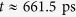 the BP enters the sample at the bottom boundary and propagates upwards until 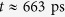 when it leaves the sample at the top boundary. In the Supplementary Video 2 the isolated skyrmion core reversal dynamics is shown.

We note that the Bloch point moves upwards in Fig. 7(d) but one may ask whether an opposite propagation direction can occur and how the Bloch point structure is going to change. We demonstrate that which of these two propagation directions will occur in the reversal process depends on the simulation parameters. The reversal mechanism simulation was repeated with increased Gilbert damping (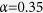 instead of 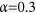) and the results showing the downwards propagation are shown in the Supplementary Section S3. We hypothesise that both reversal paths (Bloch point moving upwards or downwards) exhibit the same energy barriers and that the choice of path is a stochastic process. By analysing the results from Fig. 7(d,e) and Supplementary Fig. 6, we also observe that the change in the BP propagation direction implies the change of the BP structure since the out-of-plane magnetisation component 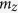 field reverses in the vicinity of BP.

## Discussion

Through systematic micromagnetic study of equilibrium states in helimagnetic confined nanostructures, we identified the ground states and reported the (meta)stability regions of other equilibrium states. We demonstrated in Fig. 2 that skyrmionic textures in the form of incomplete Skyrmion (iSk) and isolated Skyrmion (Sk) configurations are the ground states in disk nanostructures, and that this occurs in a wide 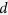
–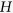 parameter space range. We have carried out similar studies for a square geometry and obtain qualitatively similar results. Of particular importance is that iSk and Sk states are the ground states at zero external magnetic field which is in contrast to infinite thin film and bulk helimagnetic samples. We note that neither an external magnetic field is necessary nor magnetocrystalline anisotropy is required for this stability. We also note in Fig. 4(c) that there is significant flexibility in the skyrmionic texture size which provides robustness for technology built on skyrmions, where fabrication of nanostructures and devices introduces unavoidable variation in geometries.

We have established that including the demagnetisation interaction is crucial for the system investigated here, i.e. in the absence of demagnetisation effects, there are other magnetisation configurations with energies lower than that of the incomplete and isolated skyrmion. We also note that the translational variance of the magnetisation from the lower side of the thin film (at 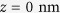) to the top (at 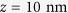) is essential for the physics reported here: if we use a two-dimensional micromagnetic simulation (i.e. assuming translational invariance of the magnetisation 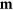 in the out-of-plane direction), the isolated skyrmion configuration does not arise as the ground state. Our interpretation is that for skyrmion-like configurations the twist of 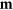 between top and bottom layer allows the system’s energy to reduce significantly while such a reduction is less beneficial for other configurations such as helices; inline with recent predictions in the case of infinite thin films^27^. Accordingly, we conclude that three-dimensional helimagnetic nanostructure models, where demagnetisation energy contribution is neglected, or the geometry approximated using a two-dimensional mesh, are not generally justified.

Because of the specific boundary conditions^28^ and the importance of including the demagnetisation energy contribution, our predictions cannot be directly applied to other helimagnetic materials without repeating the stability study. For instance, although the size of skyrmionic textures in this study was based on cubic FeGe helimagnetic material with helical period 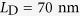, in order to encourage the experimental verification of our predictions, this study could be repeated for materials with smaller 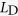. In such materials the skyrmionic core size is considerably reduced, which allows the reduction of hosting nanostructure size and is an essential requirement for advancing future information storage technologies. Similarly, the ordering temperature of simulated FeGe helimagnetic material, 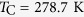
42, is lower than the room temperature, which means that a device operating at the room temperature cannot be constructed using this material. Because of that, in Supplementary Section S4, we demonstrate that our predictions are still valid if the ordering temperature of simulated B20 helimagnetic material is artificially increased to 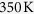.

We demonstrate in Fig. 6 that skyrmionic textures in confined helimagnetic nanostructures exhibit hysteretic behaviour as a consequence of energy barriers between energetically equivalent stable configurations (skyrmionic texture core pointing up or down). In the absence of magnetocrystalline anisotropy and if the demagnetisation energy (demagnetisation-based shape anisotropy) is removed from the system’s Hamiltonian, the hysteretic behaviour is still present, demonstrating the existence of a novel Dzyaloshinskii-Moriya-based shape anisotropy.

Finally, we show how the reversal of the isolated skyrmion core orientation is facilitated by the Bloch point occurrence and propagation, and demonstrate that the Bloch point can propagate in both directions along the out-of-plane 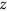-direction.

All data obtained by micromagnetic simulations in this study and used to create figures both in the main text and in the Supplementary Information are included in Supplementary Data.

## Methods

### Model

We use an energy model consistent with a non-centrosymmetric cubic B20 (P2_1_3 space group) crystal structure. This is appropriate for a range of isostructural compounds and pseudo-binary alloys in which skyrmionic textures have been experimentally observed^3^,^4^,^15^,^16^,^17^,^18^,^43^,^44^. The magnetic free energy of the system 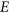 contains several contributions and can be written in the form:


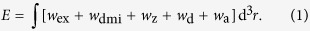


The first term is the symmetric exchange energy density 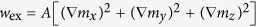 with exchange stiffness material parameter 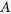, where 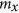, 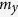, and 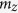 are the Cartesian components of the vector 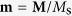 that describes the magnetisation 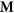, with 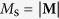 being the saturation magnetisation. The second term is the Dzyaloshinskii-Moriya Interaction (DMI) energy density 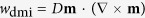, obtained by constructing the allowed Lifshitz invariants for the crystallographic class T^45^, where 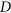 is the material parameter. The third term is the Zeeman energy density term 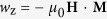 which defines the coupling of magnetisation to an external magnetic field 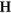. The 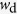 term represents the demagnetisation (magnetostatic) energy density. The last term 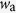 is the magnetocrystalline anisotropy energy density, and because the simulated material is assumed to be isotropic, we neglect it throughout this work. Neglecting this term also allows us to determine whether the magnetocrystalline anisotropy is a crucial mechanism allowing the stability of skyrmionic textures in confined helimagnetic nanostructures.

The Landau-Lifshitz-Gilbert (LLG) equation^36^:


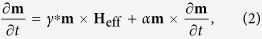


governs the magnetisation dynamics, where 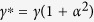, with 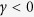 and 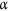 being the gyromagnetic ratio and Gilbert damping, respectively. We compute the effective magnetic field using 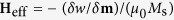, where 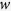 is the total energy density functional. With this model, we solve for magnetic configurations 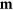 using the condition of minimum torque arrived by integrating a set of dissipative, time-dependent equations. We validated the boundary conditions by a series of simulations reproducing the results in Ref. 26,28.

### Simulator

We developed a micromagnetic simulation software, inspired by the Nmag simulation tool^46^,^47^. Unlike Nmag, we use the FEniCS project^48^ instead of the Nsim multi-physics library^46^ for the finite element low-level operations. In addition, we use IPython^49^,^50^ and Matplotlib^51^,^52^ extensively in this work.

### Material parameters

We estimate the material parameters in our simulations to represent the cubic B20 FeGe helimagnet with four Fe and four Ge atoms per unit cell^53^ and crystal lattice constant 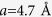
54. The local magnetic moments of iron and germanium atoms are 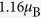 and 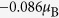
55, respectively, where 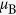 is the Bohr magneton constant. Accordingly, we estimate the saturation magnetisation as 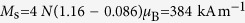, with 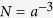 being the number of lattice unit cells in a cubic metre. The spin-wave stiffness is 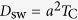
56, where the FeGe ordering temperature is 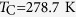
42. Consequently, the exchange stiffness parameter value is 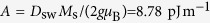 57, where 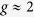 is the Landé 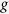-factor. The estimated DMI material parameter 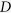 from the long-range FeGe helical period 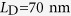
42, using 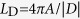
43, is 
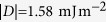
.

### Skyrmion number 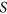 and injective scalar value 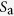


In order to support the discussion of skyrmionic textures, the topological skyrmion number^2^


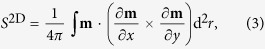


can be computed for two-dimensional samples hosting the magnetisation configuration. However, for confined systems, the skyrmion number 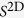 is not quantised into integers^26^,^31^, and therefore, a more suitable name for 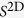 may be the “scalar spin chirality” (and consequently the expression under an integral would be called the “spin chirality density”), but we will follow the existing literature^26^,^31^ and refer to 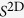 as the skyrmion number. We show its dependence on different skyrmionic textures that can be observed in confined helimagnetic nanostructures in Supplementary Fig. 2(b), demonstrating that the skyrmion number in confined geometries is not an injective function since it does not preserve distinctness (one-to-one mapping between skyrmionic textures and skyrmion number value 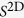). Therefore, for two-dimensional samples, we define a different scalar value


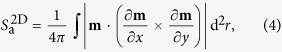


and show its dependence on different skyrmionic textures in Supplementary Fig. 2(b). This scalar value is injective and provides necessary distinctness between 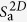 values for different skyrmionic states. In terms of the terminology discussion above regarding 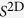, the entity 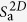 describes the “scalar absolute spin chirality”. We also emphasise that although the skyrmion number 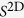 has a clear mathematical^58^ and physical^59^ interpretation, we define the artificial injective scalar value 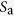 only to support the classification and discussion of different skyrmionic textures observed in this work.

Skyrmion number 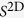 and artificially defined scalar value 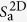, given by Eq. (3) and Eq. (4), respectively, are valid only for the two-dimensional samples hosting the magnetisation configuration. However, in this work, we also study three-dimensional samples and, because of that, we now define a new set of expressions taking into account the third dimension. The skyrmion number in three-dimensional samples 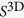 we compute using


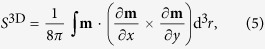


as suggested by Lee *et al*.^60^, which results in a value proportional to the anomalous Hall conductivity. Similar to the two-dimensional case, we also define the artificial injective scalar value 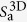 for three-dimensional samples as


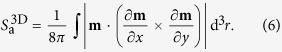


In order to allow the 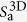 value to fall within the two-dimensional skyrmionic textures classification scheme, we normalise the computed 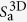 value by a constant (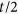, where 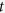 is the sample thickness).

For simplicity, in this work, we refer to both two-dimensional and three-dimensional skyrmion number and scalar value expressions as 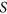 and 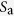 because it is always clear what expression has been used according to the dimensionality of the sample.

## Additional Information

**How to cite this article**: Beg, M. *et al*. Ground state search, hysteretic behaviour, and reversal mechanism of skyrmionic textures in confined helimagnetic nanostructures. *Sci. Rep*. **5**, 17137; doi: 10.1038/srep17137 (2015).

## Supplementary Material

Supplementary Information

Supplementary Video S1

Supplementary Video S2

Supplementary Table S1

Supplementary Table S1

Supplementary Table S1

Supplementary Table S1

Supplementary Table S1

Supplementary Table S1

Supplementary Table S1

Supplementary Table S1
