# Supplementary material for: Ground state search, hysteretic behaviour, and reversal mechanism of skyrmionic textures in confined helimagnetic nanostructures
Source: Sci Rep. 2015 Nov 25;5:17137. doi: 10.1038/srep17137 (PMC4658535; doi:10.1038/srep17137)
Supplement: Supplementary Information [file srep17137-s1.pdf]

Supplementary Information: Ground state search, hysteretic behaviour, and reversal mechanism of skyrmionic textures in helimagnetic nanostructures

Marijan Beg,[1,*] Rebecca Carey,[1] Weiwei Wang,[1] David Cortés-Ortuño,[1] Mark Vousden,[1] Marc-Antonio Bisotti,[1] Maximilian Albert,[1] Dmitri Chernyshenko,[1] Ondrej Hovorka,[1] Robert L. Stamps,[2] and Hans Fangohr[1,†]

[1]*Faculty of Engineering and the Environment, University of Southampton, Southampton SO17 1BJ, United Kingdom*
[2]*SUPA School of Physics and Astronomy, University of Glasgow, Glasgow G12 8QQ, United Kingdom*

SUPPLEMENTARY SECTION S1: INITIAL MAGNETISATION CONFIGURATIONS

The magnetisation configurations that we use as initial states for the full three-dimensional micromagnetic simulations are shown in Supplementary Fig. 1. For every point in the d–H parameter space, we relax twelve different initial magnetisation configurations. These are the five skyrmionic configurations (A, B, C, D, and E), three helical configurations (H2, H3, and H4), the uniform configuration (U), and three random magnetisation configurations (R).

Now, we introduce an approximate analytic model that helps us generate a range of physically meaningful and reproducible initial skyrmionic magnetisation configurations labelled A-E in Supplementary Fig. 1. The used DMI energy density term (see Methods section of the main text) is consistent with the helimagnetic material of crystallographic class T, and one expects a skyrmionic texture configuration with no radial spin component (chiral skyrmion) to emerge [1]. Consequently, if we consider a two-dimensional disk sample of radius R in the plane containing the x and y axes, as shown in Supplementary Fig. 2 (b) inset, an approximation of the chiral skyrmionic magnetisation texture (for $D > 0$), in cylindrical coordinates (ρ, φ, z), is

$$\begin{aligned} m_\rho &= 0, \\ m_\varphi &= \sin(k\rho), \\ m_z &= -\cos(k\rho), \end{aligned} \tag{1}$$

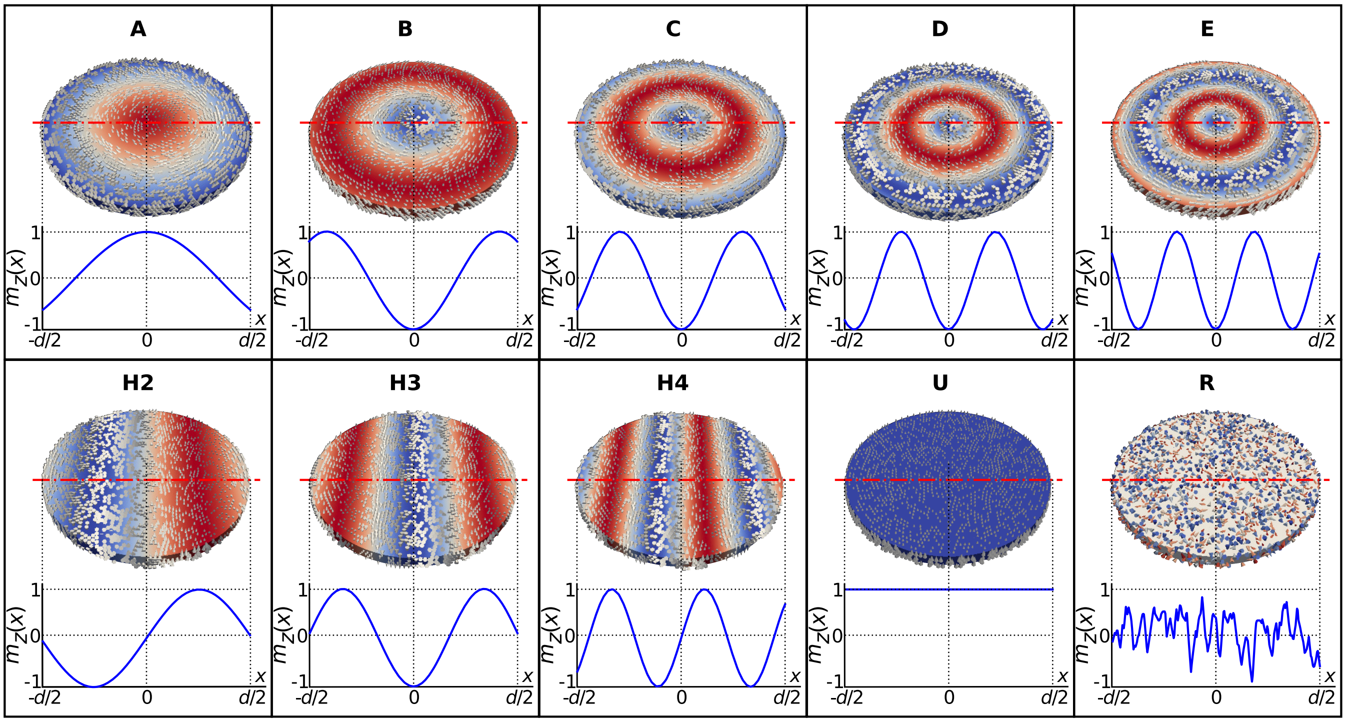

FIG. 1. **The magnetisation configurations relaxed using the full three-dimensional micromagnetic simulations.** The configurations labelled A-E correspond to the first five solutions of the approximate analytic model, whereas the three helical states with 2, 3, and 4 helical half-periods are labelled as H2, H3, and H4, respectively. The uniform out-of-plane magnetisation state is labelled as U, and an example of random magnetisation state is marked with R.

where $k = 2\pi/s$ is a measure of the skyrmionic texture size s.

An equilibrium configuration requires that the torque exerted on the magnetisation $\mathbf{m}$ vanishes at every point in sample ($\mathbf{m} \times \mathbf{H}_\text{eff} = 0$), including the boundary. The effective field functional $\mathbf{H}_\text{eff} = -(\delta w/\delta \mathbf{m})/(\mu_0 M_\text{s})$, due to only symmetric exchange and DMI energy contributions, in absence of an external magnetic field is

$$\mathbf{H}_\text{eff} = \frac{2}{\mu_0 M_\text{s}} \left[A \nabla^2 \mathbf{m} - D \left(\nabla \times \mathbf{m} \right) \right], \tag{2}$$

for any magnetisation texture. Computing this expression for the radially symmetric approximate skyrmionic texture model $\mathbf{m} = \sin(k\rho)\hat{\boldsymbol{\varphi}} - \cos(k\rho)\hat{\mathbf{z}}$ results in

$$\begin{aligned} \mathbf{H}_\text{eff} =& \frac{2}{\mu_0 M_\text{s}} \left[\left(Dk - Ak^2 - \frac{A}{\rho^2} \right) \sin(k\rho) + \frac{Ak}{\rho} \cos(k\rho) \right] \hat{\boldsymbol{\varphi}} + \\ & \frac{2}{\mu_0 M_\text{s}} \left[\left(\frac{Ak}{\rho} - \frac{D}{\rho} \right) \sin(k\rho) + \left(Ak^2 - Dk \right) \cos(k\rho) \right] \hat{\mathbf{z}}. \end{aligned} \tag{3}$$

Consequently, the torque exerted on the magnetisation $\mathbf{m}$ is

$$\mathbf{m} \times \mathbf{H}_\text{eff} = \frac{2}{\mu_0 M_\text{s}} \left[\frac{Ak}{\rho} - \frac{D}{\rho} \sin^2(k\rho) - \frac{A}{2\rho^2} \sin(2k\rho) \right] \hat{\mathbf{r}}. \tag{4}$$

Requiring the torque to vanish at the disk boundary $\rho = R$ results in the zero-torque condition:

$$g(kR) \equiv -P \sin^2(kR) - \frac{\sin(2kR)}{2kR} + 1 = 0, \tag{5}$$

where $P = D/kA$. The analysis of this condition shows that the parameter P must satisfy the inequality $P > 2/3$ in order for $g(kR)$ to have roots and, thus, a skyrmionic texture core to exist in at least metastable equilibrium. In Supplementary Fig. 2 (a), we plot the zero-torque condition as a function of kR for different values of P. Since $P = 2/3$ is the boundary case (dotted line in the plot), all plots for $P < 2/3$ have no solutions, whereas the zero-torque condition has multiple solutions if $P > 2/3$.

The skyrmionic magnetisation configurations used as initial states in the energy minimisation process, corresponding to the zero-torque condition solutions (marked A-F) for $P = 2$, we show in Fig. 2 (c). In order to support the discussion of these magnetisation configurations, we compute the skyrmion number S, which for our approximate analytic model results in $S = (\cos(kR) - 1)/2$. Its dependence on kR, presented in Fig. 2 (b) as a dashed line, shows that the skyrmion number for the skyrmionic textures in confined nanostructures is not an injective function since it does not preserve distinctness (one-to-one mapping between kR and skyrmion number value S). Therefore, a different scalar value S_a is computed and from its dependence on kR, shown in Fig. 2 (b) as a solid line, we conclude that this scalar value is injective and provides necessary distinctness between S_a values for different skyrmionic states. The details about skyrmion number S and scalar value S_a are in the Methods section of the main text.

For the helical state, which emerges as a consequence of the Dzyaloshinskii-Moriya interaction considered in this work, we expect that the magnetisation vector at any point is perpendicular to the helical propagation direction (Bloch-wall-like configuration). Consequently, if both x and y axis are in the plane of the thin-film sample and the x axis is chosen as a propagation direction, the helical magnetisation configuration in Cartesian coordinates is

$$\begin{aligned} m_x &= 0, \\ m_y &= \cos(k_\text{h} x), \\ m_z &= \sin(k_\text{h} x), \end{aligned} \tag{6}$$

where $k_\text{h} = 2\pi/\lambda$, with λ being the helical period [2].

Now, we investigate whether the helical period λ in confined nanostructures is independent on the sample diameter, and if not, what are the helical period values that can occur in our simulated system. After relaxing helical configurations and varying both the helical period and disk sample diameter (up to 180 nm), we find that all relax to a limited set of helical states with different helical periods. More precisely, the observed relaxed helical states consist of either 2, 3, or 4 helical half-periods along the disk sample diameter, including the characteristic magnetisation tilting at the boundary [3]. Thus, we define three different helical magnetisation configurations as initial states with helical periods $2d/2$, $2d/3$, or $2d/4$, where d is the disk sample diameter, and are named H2, H3, and H4, respectively. Magnetisation configurations of these states, together with their $m_z(x)$ profiles along the horizontal symmetry, are shown in Supplementary Fig. 1.

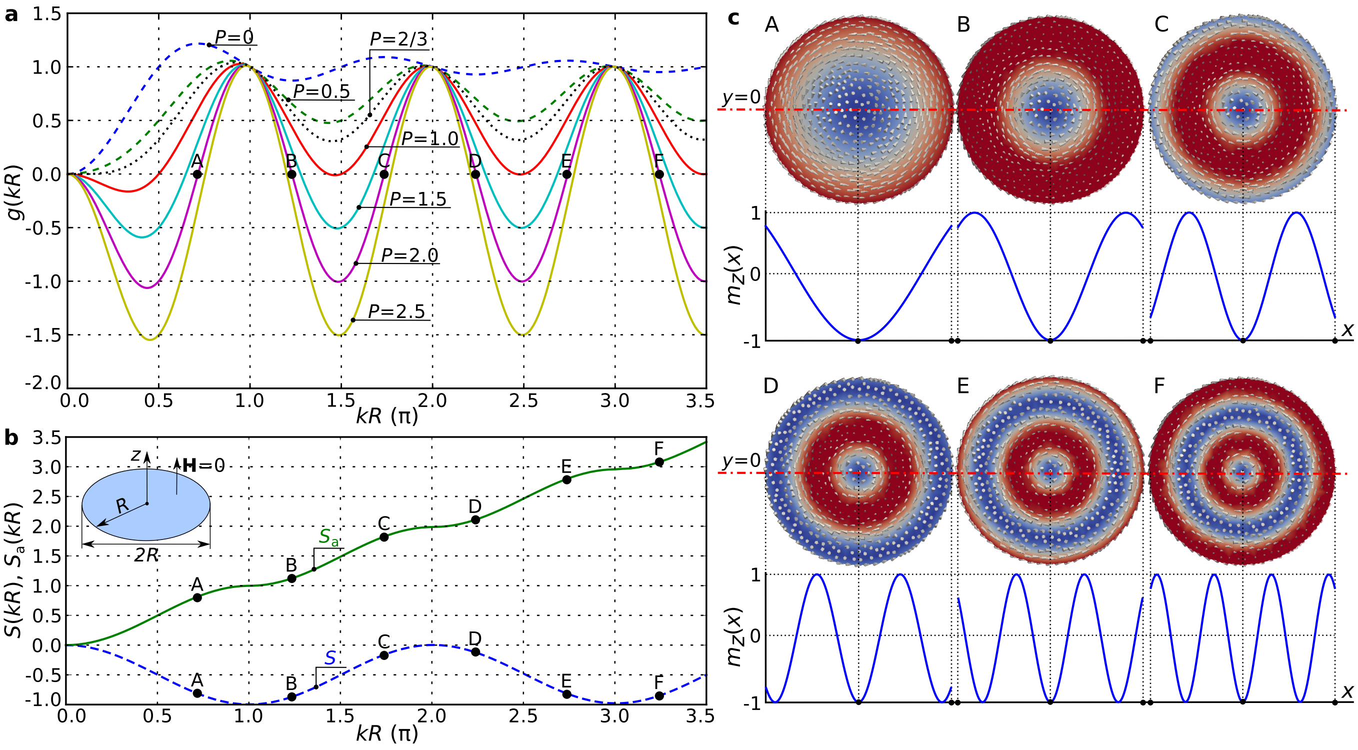

FIG. 2. **Zero-torque condition plots and magnetisation configurations corresponding to its solutions.** (**a**) The zero-torque condition $g(kR)$ plots, given by Eq. (5), for different values of $P = D/kA$. All zero-torque conditions for P smaller than the boundary case $P = 2/3$ (dotted line) have no solutions, whereas for $P > 2/3$, multiple solutions (A-F) exist. (**b**) The non-injective dependence of skyrmion number S and the injective dependence of scalar value S_a on kR. (**c**) The magnetisation configurations and the out-of-plane magnetisation component $m_z(x)$ along the horizontal symmetry line for different solutions (A-F) of Eq. (5) for $P = 2$.

In addition to the previously defined eight chiral initial states, we also use the uniform magnetisation configuration, where the magnetisation at all mesh nodes is in the positive out-of-plane z-direction, as shown in Supplementary Fig. 1 marked as U. Finally, in order to capture other equilibrium states that cannot be obtained by relaxing previously described well-defined magnetisation configurations, we also use additional three random magnetisation configurations. At every mesh node, we choose three random numbers in the $[-1, 1]$ range for three magnetisation components and then normalise them in order to fulfil the $|\mathbf{m}| = 1$ condition. An example of one random magnetisation configuration is shown in Supplementary Fig. 1 and labelled as R.

SUPPLEMENTARY SECTION S2: RELAXATION DIAGRAMS

In this section, we compute the equilibrium states at all points in the d–H parameter space obtained by relaxing well-defined initial states, introduced in the Supplementary Section S1. More precisely, we compute the equilibrium states (local energy minima) that result from a particular initial condition. This allows us to provide a systematic overview of equilibrium states, and gain additional insight about the phase space energy landscape in the studied system. We vary the sample diameter between 40 nm and 180 nm in steps of $\Delta d = 4\,\mathrm{nm}$ and the external magnetic field between $\mu_0 H = 0\,\mathrm{T}$ and $\mu_0 H = 1.2\,\mathrm{T}$ in steps of $\mu_0 \Delta H = 20\,\mathrm{mT}$. Relaxation diagrams are represented by plotting the scalar value S_a as a function of disk diameter d and applied field strength H, and Supplementary Fig. 3 shows the relaxation diagrams for skyrmionic initial configurations A-E, and Supplementary Fig. 4 shows the relaxation diagrams for helical H2, H3, H4, and uniform U initial configurations. The phase diagram of equilibrium states, shown in Fig. 1 in the main text, summarises the main results from the relaxation diagrams presented and discussed here.

We now discuss each of relaxation diagrams in Supplementary Fig. 3 and 4, where each subplot corresponds to one particular initial configuration. The scalar value S_a, as a function of disk sample diameter d and external magnetic field

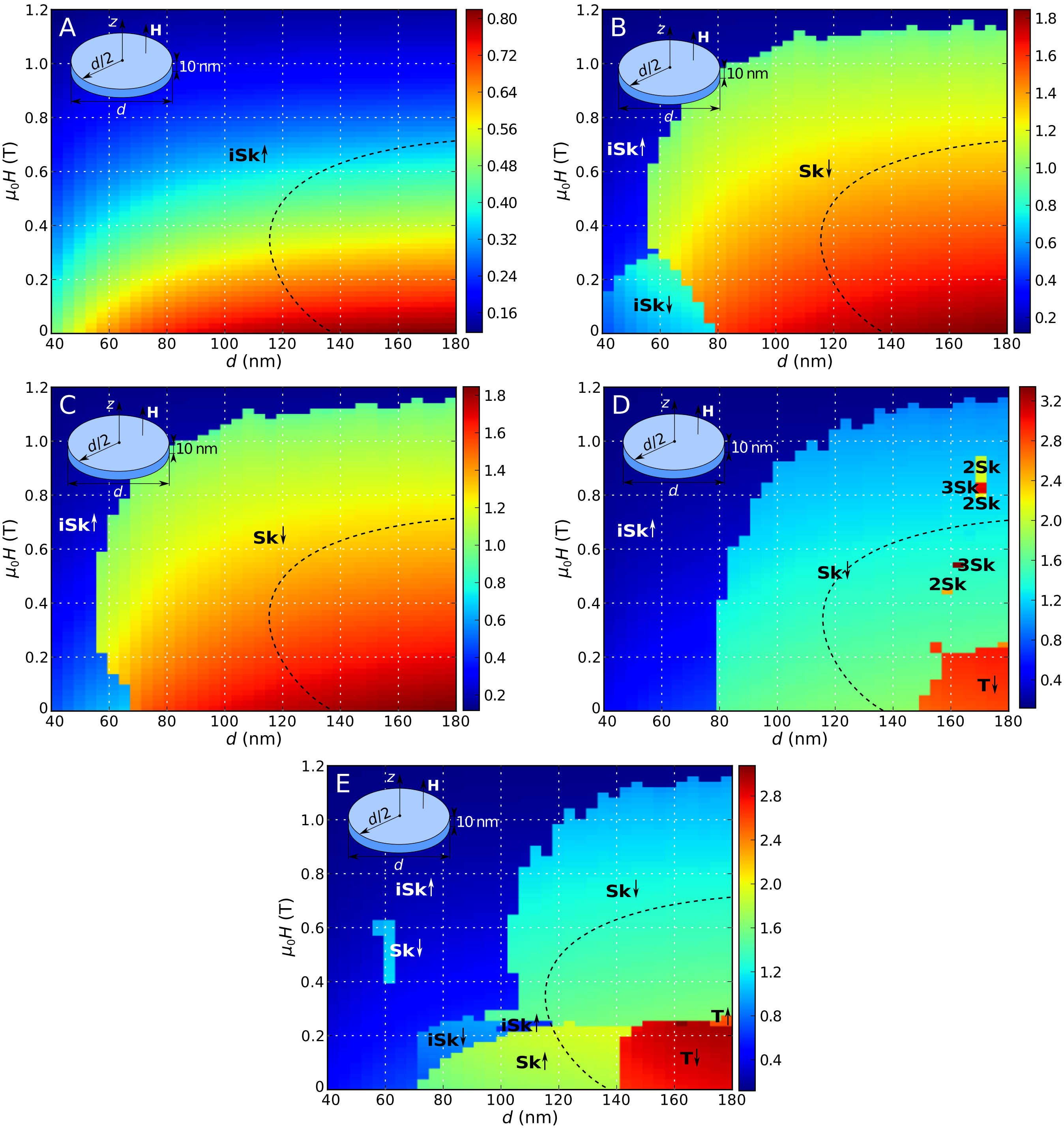

FIG. 3. **The relaxation diagrams obtained by relaxing skyrmionic initial state A-E.** The initial states correspond to the first five solutions of the analytic model and the phase diagrams are marked A-E accordingly. The relaxation diagrams are represented as the dependence of scalar value S_{a} on the disk sample diameter d and an external field H (as shown in insets).

H, computed for the final relaxed equilibrium state, i.e. local or global energy minimum, we show in Supplementary Fig. 3 (A) for the energy minimisation process that started from the skyrmionic initial configuration A (shown in Supplementary Fig. 1). The "iSk↑" label refers to the incomplete Skyrmion (iSk) magnetisation configuration with the core magnetisation pointing in the positive z-direction. An example of this state, marked with the same label, is shown in Supplementary Fig. 5. From the Supplementary Fig. 3 (A), we can see that for all examined diameters and external field values, the final relaxed configuration is the iSk↑ state. Now, this relaxation diagram is compared with

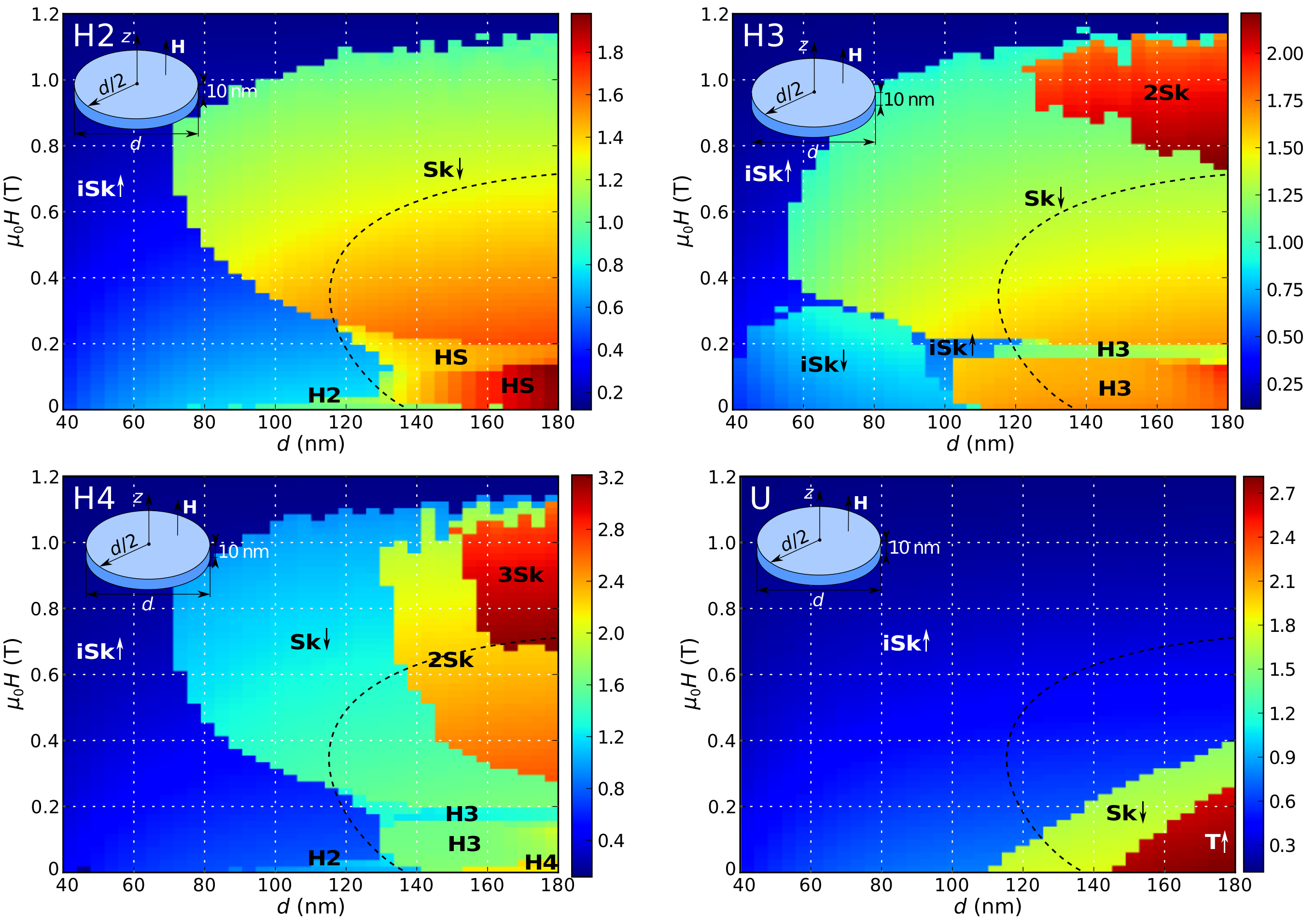

FIG. 4. **The relaxation diagrams obtained by relaxing helical and uniform initial state.** The initial states correspond to the helical states H2, H3, and H4, as well as the uniform state U, and the relaxation diagrams are marked accordingly. The relaxation diagrams are represented as the dependence of scalar value S_a on the disk sample diameter d and an external field H (as shown in insets).

the ground state phase diagram (see Fig. 2 (a) in the main text) and the boundary between two ground state (iSk and Sk) regions is shown as the dashed line in the discussed relaxation diagram. We can see that the iSk is indeed the ground state (i.e. the global energy minimum) for $d < 140\,\mathrm{nm}$, but for larger diameters the isolated Skyrmion (Sk) configuration has a lower energy, and thus the iSk configuration is only a local energy minimum.

Similarly, if the energy minimisation process is started from the initial configuration B, shown in Supplementary Fig. 1 (B), the $S_\mathrm{a}(d, H)$ for the final relaxed magnetisation state is obtained and shown in Supplementary Fig. 3 (B). The vast majority of the final configurations ($d \geq 80\,\mathrm{nm}$ and $\mu_0 H \lesssim 1.1\,\mathrm{T}$), labelled as "Sk↓", correspond to the isolated skyrmion state with the core pointing in the negative z-direction. An example of this state we show in Supplementary Fig. 5, marked with the same label. By comparison with the ground state phase diagram shown in Fig. 2 (a) in the main text (dashed line), we can see that the Sk is the ground state for large diameters $d \geq 140\,\mathrm{nm}$ and field values $\mu_0 H < 0.7\,\mathrm{T}$. However, for smaller diameters the isolated skyrmion configuration is only metastable (as the iSk configuration is the ground state). We can see that in the vicinity of $d \approx 60\,\mathrm{nm}$ and $\mu_0 H \approx 0.1\,\mathrm{T}$ parameter space point, the initial configuration B relaxes to the incomplete skyrmion state with core oriented in the negative z-direction (iSk↓), but for larger field values, configuration B falls into the iSk↑ configuration (see Supplementary Fig. 5 for detailed configurations of iSk↓ and iSk↑ states). The iSk↓ has a higher energy than the iSk↑ as the majority of the magnetisation is pointing in the direction opposite to the applied field. However, the initial configuration B is such that the core is pointing down, and there appears to be a direct energy minimisation path to the iSk↓ configuration for field values smaller than approximately $0.3\,\mathrm{T}$. For larger fields, the Zeeman energy becomes so important that the initial configuration B leads to the the iSk↑ configuration.

Supplementary Fig. 3 (C) shows that the initial configuration C suppresses the iSk↓ state completely but is otherwise

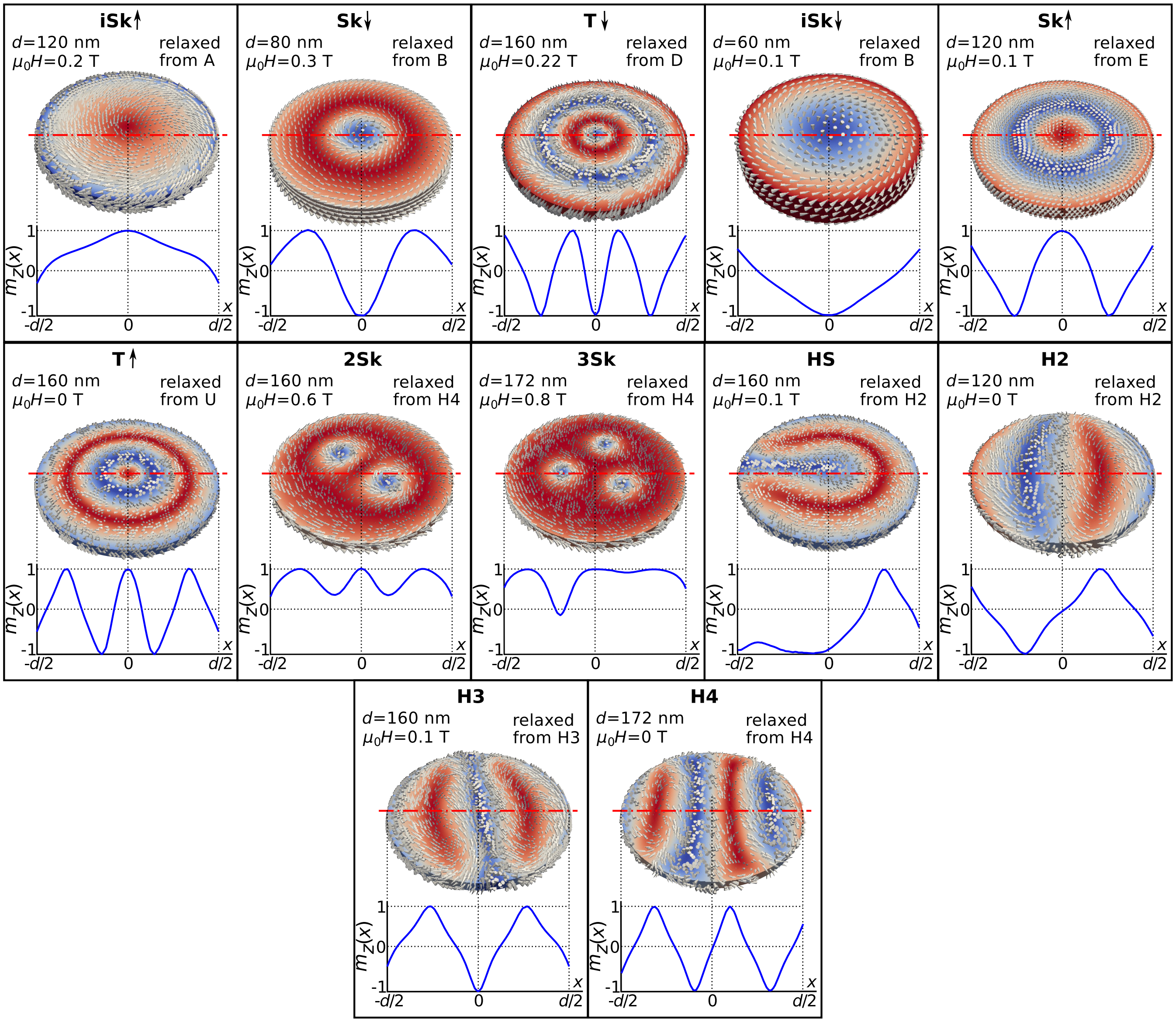

FIG. 5. **The identified equilibrium magnetisation configurations.**

similar to Supplementary Fig. 3 (B). We note in particular that the Sk configuration cannot exist for $d < 60\,\mathrm{nm}$ even if the relaxation is started from a Sk-like configuration B or C, i.e. there are no metastable isolated skyrmion states at the smallest diameters.

If the system is relaxed from the initial state D, shown in Supplementary Fig. 1 (D), a qualitative change from Supplementary Fig. 3 (B) and (C) is evident as shown in Supplementary Fig. 3 (D). In addition to the iSk↑ and Sk↓ states, there are now a number of, according to Fig. 3 in the main text, higher energy metastable states emerging as 2 or 3 skyrmions in the disk (see Supplementary Fig. 5 for detailed plots of 2Sk and 3Sk states). Furthermore, for small field values $\mu_0 H \lesssim 0.2\,\mathrm{T}$ and large diameters $d \gtrsim 152\,\mathrm{nm}$, the Target (T) equilibrium state with core orientation in the negative z-direction (T↓) arises. The T↓ state is shown in Supplementary Fig. 5.

The scalar value $S_\mathrm{a}(d, H)$ computed for final equilibrium configurations in Supplementary Fig. 3 (E) we obtained by relaxing the initial configuration E shown in Supplementary Fig. 1. The initial skyrmionic state E does not relax to 2 and 3 skyrmion configurations but allows the Sk↑ state to arise for small field values.

Supplementary Fig. 4 (H2), (H3) and (H4), show the $S_\mathrm{a}(d, H)$ for starting configurations H2, H3 and H4, respectively, as shown in Supplementary Fig. 1. All three initial configurations result in the incomplete Skyrmion configuration with core pointing up (iSk↑) for the smallest diameters as well as for largest fields. The H3 initial

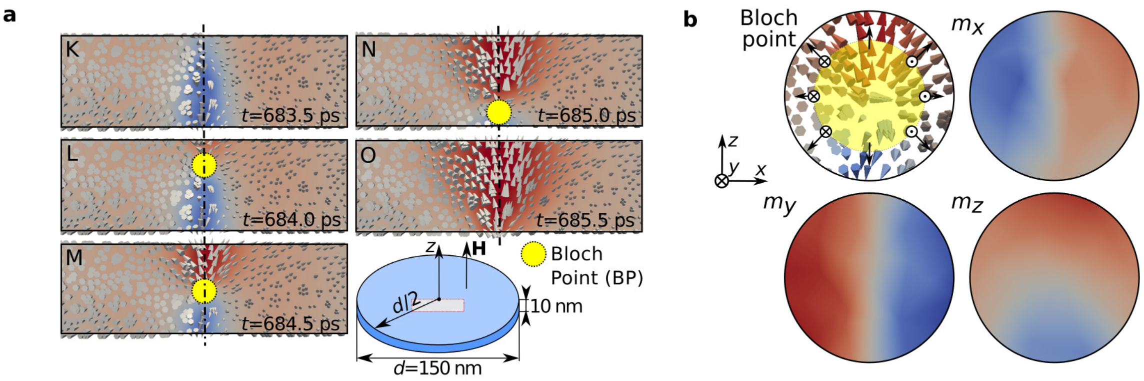

FIG. 6. **The isolated skyrmion orientation reversal in confined three-dimensional helimagnetic nanostructure with downwards Bloch point propagation direction.** (**a**) The m_z colourmap and magnetisation field in the central part of xz cross section as shown in an inset together with the position of Bloch Point (BP). (**b**) The BP structure along with colourmaps of magnetisation components which shows that the magnetisation covers the closed surface (sphere surrounding the BP) exactly once.

configuration relaxes into a configuration with two Skyrmions in the disk (2Sk) for $d > 120\,\mathrm{nm}$ and field values between 0.8 T and 1.1 T. These 2Sk configurations had appeared occasionally when starting from configuration D (see Supplementary Fig. 3 (D)). The H4 initial configuration also encourages 3 skyrmions in the disk to arise as a metastable state.

Supplementary Fig. 5 (U) shows S_a for final configurations when the simulation starts from a uniform magnetisation, pointing up in the positive z-direction. This results mostly in the incomplete Skyrmion configuration with core pointing up (iSk↑). However, we also find the Skyrmion with core pointing down Sk↓ and the Target configuration T↑ as the diameter increases and the field decreases. Supplementary Fig. 3 (A) is interesting to compare with Supplementary Fig. 4 (U): in the former, only the iSk↑ state results, presumably because from the initial state A, only the iSk↑ state is accessible in the relaxation. On the contrary, for the uniform configuration, the system finds the energy minimum for the isolated Skyrmion state Sk↓ and the Target T↑ because other energy minima can be accessed from this initial state. Fig. 5 (a) in the main text shows the relative energies of the different metastable states for $H = 0$.

SUPPLEMENTARY SECTION S3: DIFFERENT BLOCH POINT PROPAGATION DIRECTION

In order to illustrate a different direction of the Bloch Point (BP) propagation, we show a result from another skyrmion reversal. The simulation parameters are the same as in Fig. 7 of the main text, except that the Gilbert damping α is increased from 0.3 to 0.35. We show the results of isolated skyrmion core orientation reversal dynamics with modified Gilbert damping in Supplementary Fig. 6.

Now, the obtained reversal dynamics is compared with the reversal dynamics shown in Fig. 7 in the main text. From the Supplementary Fig. 6 (a), we can see that the Bloch point enters the sample at the top boundary at approximately 684 ps, then propagates downwards to the bottom boundary, where it leaves the sample at approximately 685 ps. Because of the opposite BP propagation direction, the structure of the Bloch Point changes (compare Fig. 7 (e) in the main text with Supplementary Fig. 6 (b)). More precisely, the out-of-plane magnetisation component m_z field in the vicinity of BP is changed so that for the upper half of BP $m_z > 0$, whereas in the lower half $m_z < 0$.

SUPPLEMENTARY SECTION S4: HIGHER ORDERING TEMPERATURE MATERIAL

The ordering temperature of simulated FeGe material, $T_\mathrm{C} = 278.7\,\mathrm{K}$ [4], is lower than the room temperature, which means that this material cannot be used to fabricate a device operating at room temperature. Therefore, it is important to determine how our results regarding the identified lowest energy state would change for the material with higher ordering temperature. Because no high ordering temperature helical B20 material has been reported to this day, the best we can do is to artificially increase the ordering temperature, estimate new material parameters, and repeat the study of equilibrium states.

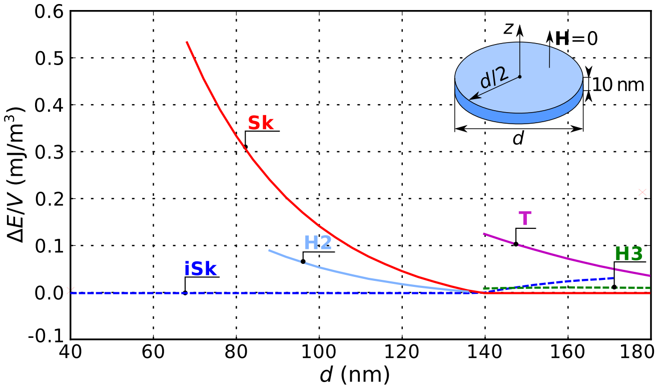

FIG. 7. **The energy density differences between all identified equilibrium states and corresponding lowest energy state as a function of disk sample diameter.** A full set of initial state configurations are relaxed for different disk sample diameter values at zero external magnetic field. The helimagnetic material ordering temperature is artificially increased to $T_\mathrm{C} = 350\,\mathrm{K}$ (above room temperature).

We increase the ordering temperature to $T_\mathrm{C} = 350\,\mathrm{K}$, and calculate new values of exchange and Dzyaloshinskii-Moriya energy constants (following the estimation described in Methods section of the main text) and obtain $A = 1.1 \times 10^{-11}\,\mathrm{J/m}$ and $D = 1.98 \times 10^{-3}\,\mathrm{J/m^2}$. Using these values, we relax the full set of initial magnetisation configurations at zero external magnetic field for different disk sample diameters. More precisely, we vary the disk sample diameter between 40 nm and 180 nm in steps of 4 nm and compute the energy density E/V of all identified relaxed equilibrium states, where V is the sample volume. After that, from the computed energy density, we subtract the energy density of the corresponding lowest energy state. We plot the calculated energy density differences of all equilibrium states as a function of disk sample diameter d and show them in Supplementary Fig. 7.

By comparing the Supplementary Fig. 7 for higher ordering temperature material with Fig. 3 in the main text, we conclude that the incomplete Skyrmion (iSk) state remains being the lowest energy state for $d < 140\,\mathrm{nm}$, whereas the isolated Skyrmion (Sk) state is the lowest energy state for $d \geq 140\,\mathrm{nm}$. However, the iSk state is not in equilibrium for disk sample diameters larger than 172 nm, which is in contrast to the FeGe material case where iSk is in equilibrium for all examined d values. Another difference is that the metastable Target (T) state is the highest energy state for the whole d range where it is in equilibrium. Finally, we do not observe H4 state (helical state with four half periods) for this high ordering temperature material.

SUPPLEMENTARY REFERENCES

---

* mb4e10@soton.ac.uk
† h.fangohr@soton.ac.uk

[1] Rößler, U. K., Bogdanov, A. N. & Pfleiderer, C. Spontaneous skyrmion ground states in magnetic metals. *Nature* **442**, 797–801 (2006).

[2] Bak, P. & Jensen, M. H. Theory of helical magnetic structures and phase transitions in MnSi and FeGe. *J. Phys. C: Solid St. Phys.* **13**, L881-L885 (1980).

[3] Rohart, S. & Thiaville, A. Skyrmion confinement in ultrathin film nanostructures in the presence of Dzyaloshinskii-Moriya interaction. *Phys. Rev. B* **88**, 184422 (2013).

[4] Lebech, B., Bernhard, J. & Freltoft, T. Magnetic structures of cubic FeGe studied by small-angle neutron scattering. *J. Phys.: Condens. Matter* **1**, 6105-6122 (1989).
